# Direct Release of Test Results to Patients Increases Patient Engagement and Utilization of Care

**DOI:** 10.1371/journal.pone.0154743

**Published:** 2016-06-23

**Authors:** Francesca Pillemer, Rebecca Anhang Price, Suzanne Paone, G. Daniel Martich, Steve Albert, Leila Haidari, Glenn Updike, Robert Rudin, Darren Liu, Ateev Mehrotra

**Affiliations:** 1 RAND Corporation, Arlington, Virginia, and Boston, Massachusetts, United States of America; 2 University of Pittsburgh School of Medicine and Graduate School of Public Health, Pittsburgh, Pennsylvania, United States of America; 3 UPMC Health System, Pittsburgh, Pennsylvania, United States of America; 4 Harvard Medical School, Boston, Massachusetts, United States of America; 5 Beth Israel-Deaconess Medical Center, Boston, Massachusetts, United States of America; 6 University of Nevada, Las Vegas, United States of America; Stellenbosch University Faculty of Medicine and Health Sciences, SOUTH AFRICA

## Abstract

An important focus for meaningful use criteria is to engage patients in their care by allowing them online access to their health information, including test results. There has been little evaluation of such initiatives. Using a mixed methods analysis of electronic health record data, surveys, and qualitative interviews, we examined the impact of allowing patients to view their test results via patient portal in one large health system. Quantitative data were collected for new users and all users of the patient portal. Qualitative interviews occurred with patients who had received an HbA1c or abnormal Pap result. Survey participants were active patient portal users. Our main measures were patient portal usage, factors associated with viewing test results and utilizing care, and patient and provider experiences with patient portal and direct release. Usage data show 80% of all patient portal users viewed test results during the year. Of survey respondents, 82.7% noted test results to be a very useful feature and 70% agreed that patient portal has made their provider more accessible to them. Interviewed patients reported feeling they should have direct access to test results and identified the ability to monitor results over time and prepare prior to communicating with a provider as benefits. In interviews, both patients and physicians reported instances of test results leading to unnecessary patient anxiety. Both groups noted the benefits of results released with provider interpretation. Quantitative data showed patient utilization to increase with viewing test results online, but this effect is mitigated when results are manually released by physicians. Our findings demonstrate that patient portal access to test results was highly valued by patients and appeared to increase patient engagement. However, it may lead to patient anxiety and increase rates of patient visits. We discuss how such unintended consequences can be addressed and larger implications for meaningful use criteria.

## Introduction

A key goal of the HITECH Act is using the EHR to engage patients and families in their care.[1] To reach this goal, meaningful use criteria provide specific objectives for EHRs, such as providing patients the ability to message providers electronically and to view their health information online. One important example is direct release, whereby test results are released directly (immediately or with a delay of several days) to patients on a patient portal, without health care providers as intermediaries. While meaningful use criteria and other federal policy initiatives encourage direct release,[1] best practices for direct release and the impact of direct release on patients and their providers remain unclear.[2] In spite of this, patient portal usage is increasing nationally,[3, 4] and the medical and policy community is working to understand how patients use these data and the impact it has on care.

There are several potential benefits and harms of direct release. Proponents argue that direct access will lead to more informed patients who are more engaged and better managers of their care.[5] Another potential benefit is improving patient safety. One study found that 8–26% of abnormal test findings are not followed up in a timely manner.[6] Direct access may allow patients to pursue appropriate follow-up. A major concern is that direct access will lead to unnecessary patient anxiety from test results that are hard to interpret.[7] This anxiety may lead patients to contact their providers, increasing phone calls and potentially office visits. At least one study has linked patient access to medical records with increased utilization.[8]

To evaluate these potential benefits and harms, we examined the impact of direct release in one large health care system in which patients with patient portal access are automatically given access to their test results. We used a mixed methods approach, including medical record review, patient portal data, and interviews to evaluate the potential impact of direct release of medical tests to EHRs, on patient safety, anxiety, and utilization.

## Materials and Methods

### Ethics Statement

This study was approved by the University of Pittsburgh Internal Review Board. As many of the qualitative interviews were conducted over the phone, study participants gave verbal consent in response to a standardized statement of informed consent developed for this study. The IRB approved this consent procedure for the study.

### System Description

UPMC is a large integrated delivery system in Western Pennsylvania with 20 hospitals and over 400 outpatient practices. In its outpatient practices, UPMC uses Epic (Verona, WI) and it uses Epic MyChart (branded as MyUPMC) as its patient portal. The patient portal was started in 2007 and as of May 2013, ~160,000 people had signed up for the patient portal. Beyond the ability to view test results, the patient portal also allows users other functionalities such as entering health data (e.g. blood pressure readings), requesting refills of medications, viewing upcoming appointments, and secure messaging with physicians and other providers.

### System for Providing Direct Access to Test Results

Tests done within UPMC or results that are transmitted electronically from diagnostic testing centers (e.g., Quest) are able to be viewed through the patient portal. Over time UPMC has included the following types of test results: laboratory, radiology, pathology, cardiology, pulmonary, and gastroenterology procedures.

Within the patient portal, test results are presented to the patient exactly as they are shown to the ordering physician in the electronic medical record. One notable exception is imaging, for which films and image files are too large, and the patient only sees the written interpretation by the radiologist.

After the ordering physician views the results, he or she can release the test results to the patient. With physician-release, the provider can also send a message explaining his or her interpretation of the results alongside electronically released test results. However, this is not required of providers. If the provider does not release the result, the test results are auto-released to the patient (regardless of whether a clinician has viewed the results) automatically within a specified timeframe after the test results enter the EHR. The auto-release default at UPMC is 48 hours; however, UPMC has let individual departments make the final determination about which tests get released. For example, the auto-release time of complicated tests (e.g, PET or nuclear scans) is greater than 48 hours and some extremely sensitive tests (e.g., HIV) are never auto-released.

With all results, the patient receives an email stating that there is a non-urgent message related to tests available to them in the patient portal and that their provider would contact them in the case of anything urgent. Patients can log into the patient portal using a personal computer or a mobile app on a smart phone, and view their result. For many results, there are ‘additional information’ links provided with the results which provide background on the test and what abnormal results could mean. The additional information is based on a database of health information. If patients have had the test before, they can view historical results for the test, in text or graphical form.

### Overview of Mixed Method Approach

We use a mixed methods approach to capture different viewpoints on the impact of direct patient access to test results. We use data from 4 distinct sources:

1. Patient portal usage data
2. EHR data on (patient) test views, office visits, and other forms of physician-patient communication
3. Qualitative interview data of patients and providers; and
4. Survey data from patient portal users’ regarding their experience with direct access test results

### Quantitative Methods

#### Patient portal usage data

We identified all users of the patient portal during a one-year period (4/1/2011 to 3/31/2012) and the functionalities used during each login. Over a single year, approximately three-quarters of patient portal account holders used the patient portal at least once.

#### EHR data on types of tests viewed and patient utilization

Among patients recently enrolled in the patient portal, we identified new users who had a test result released for the first time between November and December 2011. We examined whether the patients viewed the results, which tests were released, and whether they were physician-released or auto-released. We compared viewers and non-viewers of test results according to demographic status (age, gender, race), number of medical conditions in the active problem list, and test status.

To assess the impact of direct release on patient utilization, we used a difference-in-difference analysis comparing utilization before and after patient portal access to test results among those who did and did not view normal test results when they were available via the patient portal.[9] The premise underlying this analysis is that if patients’ illness is relatively stable and the tests were all normal then the major difference in utilization between the two periods is driven by direct release. Specifically, among the population of first-time test results, we calculated the proportion of patients who had office and telephone encounters in a period before access to test results [Jan-Mar 2011,almost a year before] and period after access to test results [Jan-Mar 2012, three-month period after their first viewing of test results]. We chose not to look at utilization during the time period of test release because we did not want to capture utilization that triggered the ordering of the test. To estimate the impact we used a regression framework for difference-in-difference analyses where the unit of analysis is the individual and we include variables for time period (pre/post), whether the individual used viewed test results (yes/no), and an interaction term for time-period/viewed test results. The impact of direct release and the associated p-value are based on the interaction term.

### Qualitative Methods

#### Qualitative interview data

We conducted qualitative interviews with physicians and patients. The interviews were conducted via telephone by the study authors and were audio recorded and transcribed. The physicians were part of an EHR advisory panel of practicing primary care physicians. We focused on their experiences with direct release, their perception of its impact, and systems within their practices related to test results (e.g., Does the physician or nurse deliver abnormal tests?).

We also interviewed patients with a patient portal account who had either an abnormal Pap result or any HbA1c result from the portal either by auto-release or their physician manually released the result. These two test results were chosen because Pap results involve relatively complex provider language and patients are likely to have strong psychosocial reactions to the findings.[10, 11] The HbA1c is used as a marker of diabetes control, and was chosen because it represents a typical chronic condition. Given that one goal of direct release of test results is to engage patients, we thought the experience of diabetics and HbA1c would provide an opportunity to assess the linkages between patient portal usage and patients’ perceptions of their engagement.

We randomly selected patients who had received HbA1c/PAP results and sent an email invitation. 13 interview participants were offered a $25 gift card for participation in a 1-hour discussion. The interviews focused on patient experiences (e.g., positive and negative experiences), emotional responses (e.g. anxiety), and preferences (e.g. preference for seeing results whether the provider has reviewed the result or not). We explored their general experience with receiving test results in general and how they were first likely to receive those results (i.e., from their physician or the portal). We coded the interview data using Atlas Ti software, and generated preliminary codes using grounded theory methodology. Given the size of the study, we did not use a multiple coder system. However, coding was developed (in accordance with grounded theory) in an iterative fashion, and with full team participation.

#### Survey data

We sent a quality assurance survey to users of patient portal about their experience in November-December, 2012. All patient portal users that logged in at least 6 times in the prior year (the most active half of users) were invited via email to access an internet survey. The survey focused on their experiences with the patient portal, what aspects they valued most, and functionalities they would like to add to the patient portal. No incentive was given for participation. Of the approximately 54,000 invited to complete the questionnaire surveyed, 6,368 (12%) responded.

## Results

### Use of Test Results

In the two-month period studied, there were 77,901 test results released to 14,441 patients with patient portal accounts. The mean number of released tests per patient was 5.4 (11.5 SD). Of all patients with a test result, 43.3% of patients had at least one abnormal value. The mean age of patients was 51.8 (15.3 SD). The sample was mostly white (91.4%) and had a slight preponderance of males (54.8%) [Table 1].

**Table 1:** Characteristics of Patients using a Patient Portal during the 2 month study period.

	All Patients, n = 14,441	Test Viewers, n = 8,486	Non-Viewers, n = 5,955
Viewed Test %	58.84	—	—
Abnormal Result %	43.3	47.7	37.1*
Race, white %	91.4	91.5	91.4
Gender, male %	54.8	54.3	55.7
Age, mean (SD)	51.8 (15.3)	50.9 (15.2)	53.0 (15.5)*

* p < 0.001

Of test results released to patients, 58.8% were viewed by a patient via the patient portal. Viewers and non-viewers of released tests did not differ in gender or race, but viewers were younger (50.9 vs. 53.0, p < .001). Of the test results, 29.6% were direct release and the remainder auto-release.

### Patient Engagement and Safety

One potential benefit of direct patient access is improving patient engagement. Patients in our qualitative interviews felt a strong sense of ownership of their results and nearly all thought that patients should have direct access. One respondent noted, “The more informed you are, the better you can handle and control your illnesses and diseases.” Patients with diabetes valued the ability to monitor their results over time. One respondent commented, “Some people get labs and tests done regularly, often the same tests … They tend to know how to interpret results.”

Patients believed that direct access gave them time to review results and prepare questions prior to a provider communication, “Getting results first … gave [one respondent] time to do research and come to appointment prepared. It’s hard to do that on the spot, when getting results during the appointment.” Further, some noted practical issues, such as the inability to write down complete test results in a phone conversation which leads to a less complete understanding of their health.

patient portal users’ survey responses and patient portal use patterns underscore patients’ interest in direct access to test results. Of survey respondents, 82.7% noted test results to be a very useful feature and 70% responded that they somewhat or completely agree with the statement that patient portal has made their provider more accessible to them. The average patient portal user logged-on 13 times in a given year and almost half (48%) of all log-ins involved viewing of test results. Of all users of the patient portal, 80% viewed tests results at some point during the year.

One of the goals of direct patient access to test results is to improve patient safety by increasing the likelihood that patients are aware of abnormal results requiring follow-up. The physicians we spoke with were unsure if patient portal access to test results improved patient safety. However, one noted that the value of even a small number of ‘missed’ results “caught” by patient portal use would be worth a lot. In our 13 qualitative patient interviews, two patients reported instances in which the patient portal improved the quality of their health care. In the first instance, a patient saw an abnormal Pap test result and contacted her provider after 3 days; the provider apologized and set up follow-up testing for the patient. In the second instance, a patient received results suggesting a critical and time-sensitive abnormality in blood work and called her physician.

### Patient Anxiety and Association of Auto-release and Utilization

There is a concern that providing patients access to their test results will increase patient anxiety and confusion resulting in additional patient contact with their clinician, excess utilization and costs. Many physicians we spoke with were concerned about patient anxiety resulting from patient portal test release. At the extreme, one physician noted cynically that only patients who were generally anxious signed up for the patient portal and availability of the test results only increased that anxiety. Several providers described experiences in which patients contacted them for abnormalities that were clinically insignificant, increasing the clinician’s workload. Patients also reported instances in which test results drove unnecessary anxiety. In one example, a patient received an abnormal Pap test on a Friday evening, and believed herself to have cancer, which resulted in significant anxiety. It wasn’t until the following Monday that her clinician was able to reassure her. In another example, a patient noted that there was an “H” (indicating “high”) by a blood sugar result, and he was concerned about being prediabetic until speaking with his clinician.

Interviewed patients and physicians noted important distinctions between provider-released versus auto-released test results, and results accompanied by physician interpretation versus those that had no accompanying interpretation. Some physicians perceived that quick interpretations of the results eliminated patient anxiety. One patient stated “I did not know what some of the numbers were, or what they meant or would represent, and there was no explanation for the numbers… All I needed was the letter saying nothing was abnormal.” Another patient reported that “viewing her results when it looks like the physician hasn’t viewed them yet [increased the chances of her] having to call to ask about a result that may be abnormal.”

This was echoed in our quantitative analyses (see Table 2). Using a difference-in-differences technique, we compared those who viewed any type of test results (abnormal or normal, manually released or auto-released) to those who did not view test results before and after automated test release became available. Viewing test results was associated with an increase in the number of patients with an office or telephone visit (3.7% and 4.6%, respectively (p < .001), Table 2). Our results were similar when we limited the analyses to only those who had only normal test findings (3.0% and 4.0%, respectively) and limited it even further to only thosewith normal test findings released via auto-release (3.9% and 4.9%, respectively, p < .001). However, if physicians manually released test results, the increases in office and telephone contact were smaller and not statistically different (1.7% and 2.3%, for office visits and phone calls, respectively).

**Table 2:** Comparison of Utilization of Office Visits and Telephone Calls Before and After Direct Release.*

	Any Office Visits				Any Telephone Calls			
	Pre-PHR access to test results (Jan-Mar 2011) %	PHR access to test results (Jan-Mar 2012) %	Differ-ence	Difference in differences	Pre-Direct Release (Jan-Mar 2011) %	Post-Direct Release (Jan-Mar 2012) %	Differ-ence	Difference in differences
**All Patients, n = 14,441**								
Viewers*	12.0	19.8	7.8	3.7 ^†^	9.4	18.2	8.8	4.6 ^†^
Non-Viewers	10.5	14.6	4.1		8.8	13	4.2	
**Patients Whose Test Results Were Normal, n = 8,183**								
Viewers	10.8	17.3	6.5	3.0 ^†^	7.9	15.5	7.6	4.0 ^†^
Non-Viewers	9.3	12.8	3.5		7.8	11.4	3.6	
**Patients Whose Test Results Were Normal and Tests Auto-Released, n = 5,667**								
Viewers	9.1	16.5	7.4	3.9 ^†^	7.0	15.8	8.8	4.9 ^†^
Non-Viewers	8.1	11.6	3.5		6.9	10.8	3.9	
**Patients Whose Test Results Were Normal and Tests Physician-Released, n = 2,376**								
Viewers	13.5	18.8	5.3	1.7 (p = 0.58)	9.5	14.7	4.9	2.3 (p = 0.08)
Non-Viewers	12.8	16.4	3.6		10.3	12.9	2.6	

* Viewers are patients who used the PHR to view test results available to them at some point between January and March 2012

^†^ p < 0.001

## Discussion

This study’s mixed methodology enabled us to assess actual patient behavior (quantitatively), while exploring (qualitatively) the mechanisms that may be driving that behavior. We find that patients greatly value the ability to view their test results. Consistent with the prior literature,[12–14] viewing test results drove almost half of patient portal use in the health system under study, and many patients felt strongly they *should* have access to this information. Another benefit discussed in patient interviews was the potential to avert safety events. One physician noted that preventing even a small number of safety events would offset substantial system investment. However, our results also demonstrated that direct release sometimes leads to unnecessary anxiety and may increase physician workload via more office visits and telephone calls. This is consistent with prior work on the impact of patients viewing online notes.[15, 16]

Federal policy is strongly promoting patient access to laboratory test results.[17] Given the current consensus to allow patients to access their test results via the patient portal, our results are most helpful in highlighting how systems can be improved to guide best practices for direct release of test results.[2, 18]

Our findings suggest that direct release systems can be improved in two major ways. First, facilitating sharing of clinician interpretation (e.g., by creating ways clinicians can attach a message such as “I have reviewed this result and everything appears fine.” may help to address anxiety and health care utilization associated with receipt of uninterpreted test results).[17, 19] Health systems and new meaningful use criteria could make it clear that addition of such provider interpretation within two days of test result is the expected norm for patient care.[20]

Second, systems could be developed to make it easier for patients to interpret results themselves. The system studied displayed a basic presentation style and patients viewed the “raw data” presented to physicians. More advanced presentations of test results that focus on easier-to-interpret graphical presentations may decrease anxiety.[21] Other advances could include automated interpretation of the test results. Common tests with complex language such as Pap smears or colonoscopies could automatically be summarized into a level more easily interpreted by a lay person. These efforts are particularly important given that the current population of patient portal users is likely more educated than the average patient.[22] As patient portal usage becomes more widespread, information will need to be accessible to a much broader population.

As with all policy implementation, and particularly with HIT, there are also many logistic design issues which can have implications for provider adoption or patient care. As an example, our interviews with patients suggested that auto-releasing test results on Friday evenings was particularly problematic, as there was a long delay before a patient could reach their provider. Attention to detail in the design of system implementation could lead to better outcomes.

There are several key limitations of our findings. Our results are limited to a single health system which has developed its own system for sharing test results via a patient portal. The response rate of the patient survey was very low (~12%) though we note that in our mixed methods approach the survey findings were echoed in our interviews and quantitative analyses. We focused on diabetes and abnormal PAP results as illustrative studies, but we recognize that they are not generalizable to all types of tests. Our analyses of the impact of releasing test results directly to patients on their healthcare utilization could be biased if the increase in utilization we observe is driven by changes in patients’ condition rather than access to their test results.

There is no doubt that the use of a secure patient portal to communicate results from provider to patient is a clear step towards meeting the patient communication standards of meaningful use criteria compliance. However, like all policy interventions, meaningful use criteria are likely to have both positive and negative effects. Evaluations of meaningful use criteria will help drive criteria improvement are critical to maximizing the positive benefits and minimizing the negative consequences of such initiatives.

## Conclusions

Our findings demonstrate that patient direct access to test results was highly valued by patients and appeared to increase patient engagement. However, it may lead to patient anxiety and increase rates of patient visits. Such unintended consequences should be addressed in future iterations of the federal government’s meaningful use criteria and highlight the importance for health systems to integrate physicians into the release processto mitigate these consequences.
